# Glucose uptake regulation in *E. coli *by the small RNA SgrS: comparative analysis of *E. coli *K-12 (JM109 and MG1655) and *E. coli *B (BL21)

**DOI:** 10.1186/1475-2859-9-75

**Published:** 2010-09-28

**Authors:** Alejandro Negrete, Weng-Ian Ng, Joseph Shiloach

**Affiliations:** 1 Biotechnology Core Laboratory, NIDDK, NIH, Bethesda, MD USA; 2 Department of Chemical and Biomolecular Engineering, University of Maryland, College Park, MD USA

## Abstract

**Background:**

The effect of high glucose concentration on the transcription levels of the small RNA SgrS and the messenger RNA ptsG, (encodin*g *the glucose transporter IICB^Glc^), was studied in both *E. coli *K-12 (MG1655 and JM109) and *E. coli *B (BL21). It is known that the transcription level of *sgrS *increases when *E. coli *K-12 (MG1655 and JM109) is exposed to the non-metabolized glucose alpha methyl glucoside (αMG) or when the bacteria with a defective glycolysis pathway is grown in presence of glucose. The increased level of sRNA SgrS reduces the level of the ptsG mRNA and consequently lowers the level of the glucose transporter IICB^Glc^. The suggested trigger for this action is the accumulation of the corresponding phospho-sugars.

**Results:**

In the course of the described work, it was found that *E. coli *B (BL21) and *E. coli *K-12 (JM109 and MG1655) responded similarly to αMG: both strains increased *SgrS *transcription and reduced *ptsG *transcription. However, the two strains reacted differently to high glucose concentration (40 g/L). *E. coli *B (BL21) reacted by increasing *sgrS *transcription and reducing *ptsG *transcription while *E. coli *K-12 (JM109 and MG1655) did not respond to the high glucose concentration, and, therefore, transcription of *sgrS *was not detected and ptsG mRNA level was not affected.

**Conclusions:**

The results suggest that *E. coli *B (BL21) tolerates high glucose concentration not only by its more efficient central carbon metabolism, but also by controlling the glucose transport into the cells regulated by the sRNA SgrS, which may suggest a way to control glucose consumption and increase its efficient utilization.

## Background

The currently preferred method for producing recombinant proteins from *E. coli *is to grow the bacteria to high cell density and consequently increase the volumetric productivity [1]. Growing the bacteria to high cell density is accomplished mainly by proper supply of dissolved oxygen and carbon source, usually glucose [2]. While implementing high density growth strategies, it was established that the central carbon metabolism in *E. coli *B (BL21) and *E. coli *K-12 (JM109) operates differently, especially when the bacteria are exposed to high glucose concentrations [3-6]. These differences are, at least in part, the result of a functioning glyoxylate shunt and high active gluconeogensis pathways in *E. coli *B (BL21) compared with *E. coli *K-12 (JM109). As a result of these activities, *E. coli *B (BL21) tolerates higher glucose concentrations, produces less acetate, and grows to higher OD than *E. coli *K-12 (JM109) [5].

In the last several years, attention has been directed towards the small non-coding RNA (sRNA) in *E. coli*, and so far more than 70 sRNAs have been identified [7]. These molecules of approximately 40-400 nucleotides in length are involved in the regulation of pathways in response to stress conditions and environmental changes. The regulation occurs by altering translation or stability of mRNA and specific proteins through base pairing with target mRNAs or by binding to the proteins [8,9].

One of the stress conditions studied was phosphosugars accumulation; in response to this accumulation, the mRNA ptsG, responsible for encoding the major glucose transporter, IICB^Glc^, was degraded [10-12]. The degradation occurs when the cells were exposed to the non-metabolizable glucose analog alpha methyl glucoside (αMG) [13], and also in response to the accumulation of glucose-6-phosphate (G6P) or fructose-6- phosphate in *E. coli *W3110 as a result of mutations in the glycolytic pathway [11,12].

The molecule responsible for mRNA ptsG degradation was found to be the small RNA, SgrS, which was induced when the bacteria was exposed to αMG or when mutation in the glycolytic pathway caused G6P accumulation [11,13]. The sRNA SgrS that caused the degradation of mRNA ptsG was also found to encode SgrT, a 43 amino acids polypeptide. This polypeptide rescues cells growing on LB media in the presence of αMG by inhibiting glucose transport even without mRNA degradation [14]. This phenomenon indicated physiological redundancy in response to phosphosugar accumulation. We suggest that the SgrS-ptsG mechanism may be activated not only in response to non-metabolized glucose analog or to defective glycolytic enzymes, but also in response to high glucose concentration. Therefore, we decided to evaluate the response to high glucose in both *E. coli *B (BL21) and *E. coli *K-12 (JM109), two strains that are known to be different in their behavior when they are exposed to high glucose concentration [5]. If the assumption that acetate accumulation in *E. coli *grown at high glucose correlates with *sgrS *transcription and biosynthesis of the glucose transporter is correct, then it might be possible to regulate the acetate excretion by controlling the *sgrS *transcription.

## Results

### Transcription of *sgrS *and *ptsG *in *E. coli *B (BL21) and *E. coli *K-12 (JM109, MG1165) growing in shake flasks in the presence of 20 g/L glucose or 10 g/L αMG

**Figure 1:**
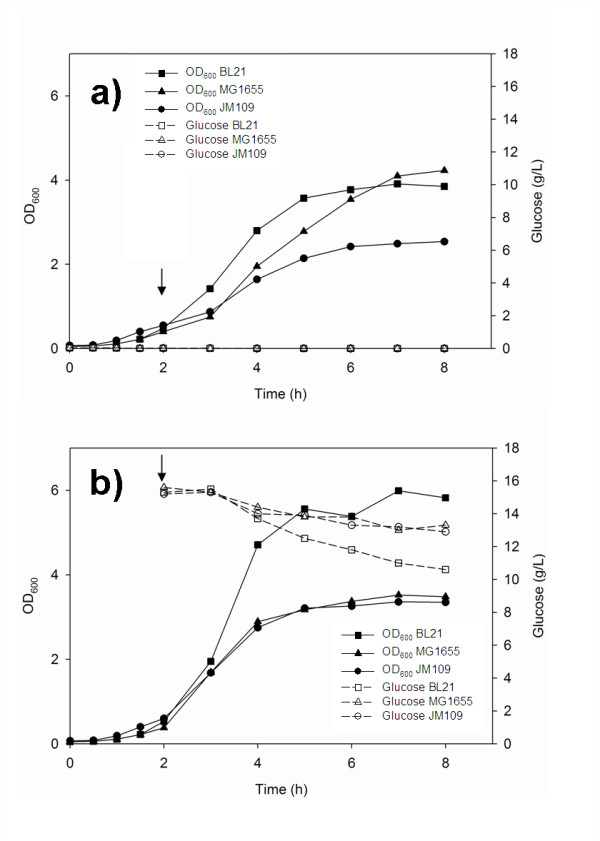
**Growth parameters of *E. coli *in shake flasks**. Growth of *E. coli *in shake flask on: a) 10 g/L α-methyl glucoside, b) 20 g/L Glucose. Arrow indicates glucose or αMG addition.

**Figure 2:**
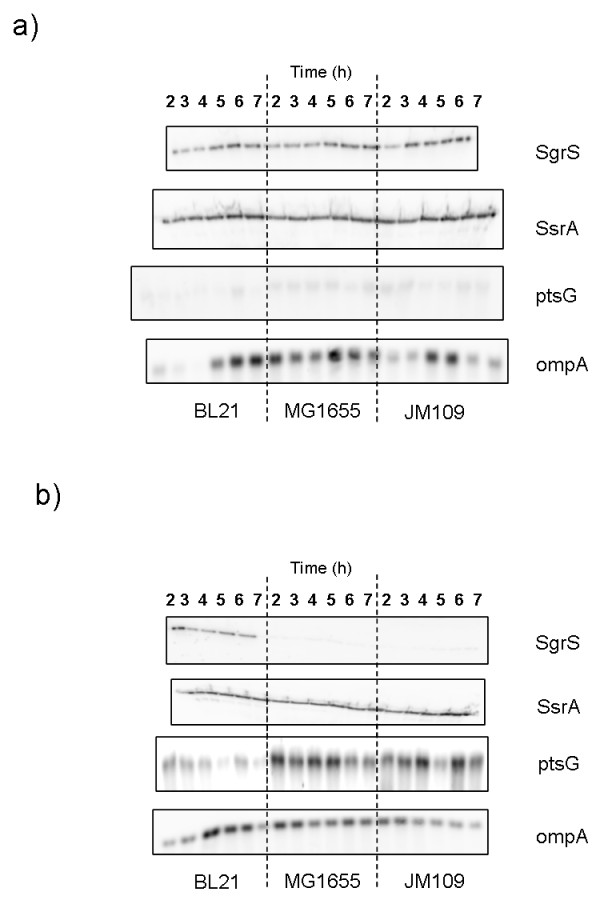
**Transcription levels of sRNA and mRNA from *E. coli *growing in shake flasks**. Transcription levels of *sgrS*, *ssrA*, *ptsG*, and *ompA *in *E. coli *B (BL21), *E. coli *K-12 (MG1655), and *E. coli *K-12 (JM109) grown in shake flask a) 10 g/L α-methyl glucoside b) 20 g/L glucose.

Previous studies of the role of SgrS and ptsG in *E. coli *metabolism were performed in shake flasks cultures; therefore the first phase of the present work was the evaluation of the differences between *E. coli *K-12 (MG1655 and JM109) and B (BL21) in shake flasks. The bacterial growth and the transcription of *sgrS *and *ptsG *in *E. coli *B (BL21) and *E. coli *K-12 (MG1655 and JM109) on LB medium in presence of αMG or glucose in shake flasks are shown in Figures 1 and 2. After 2 hours growth 20 g/L glucose or 10 g/L αMG were added to the media (Addition indicated by the arrow in Figure 1). In the presence of 10 g/L αMG *E. coli *K-12 (JM109) grew to an OD_600 _of 2, while the other two strains grew to an OD_600 _of 4 (Figure 1a). When the three strains were grown in 20 g/L glucose, the maximum OD_600 _value obtained was 6 for *E. coli *B (BL21) and 4 for the other two strains (Figure 1b). In the presence of glucose, cells reached stationary phase after 3 hours growth, which is the result of the decrease in pH. The pH at the end of the run was around 4.5 for all strains. After six hours growth, the glucose concentration in the media of the three strains was around 10 g/L.

The corresponding *sgrS *and *ptsG *transcriptions following the addition of glucose or αMG are shown in Figure 2. When αMG was added to the medium, sRNA SgrS was observed in all three strains at all time points (Figure 2a), while mRNA ptsG appeared as faint bands. When the three strains were grown at a glucose concentration of 20 g/L, only *E. coli *B (BL21) showed transcription of *sgrS *(Figure 2b) whereas mRNA ptsG was detected in all strains. But when compared with *E. coli *K-12 (JM109 and MG1655) lower transcription of *ptsG *was observed in *E. coli *B (BL21). The internal controls SsrA and ompA were observed as uniform set of bands in all cases.

### Transcription of *sgrS *and *ptsG *in *E. coli *B (BL21) and *E. coli *K-12 (JM109, MG1165) growing in bioreactor in the presence of 40 g/L glucose

**Figure 3:**
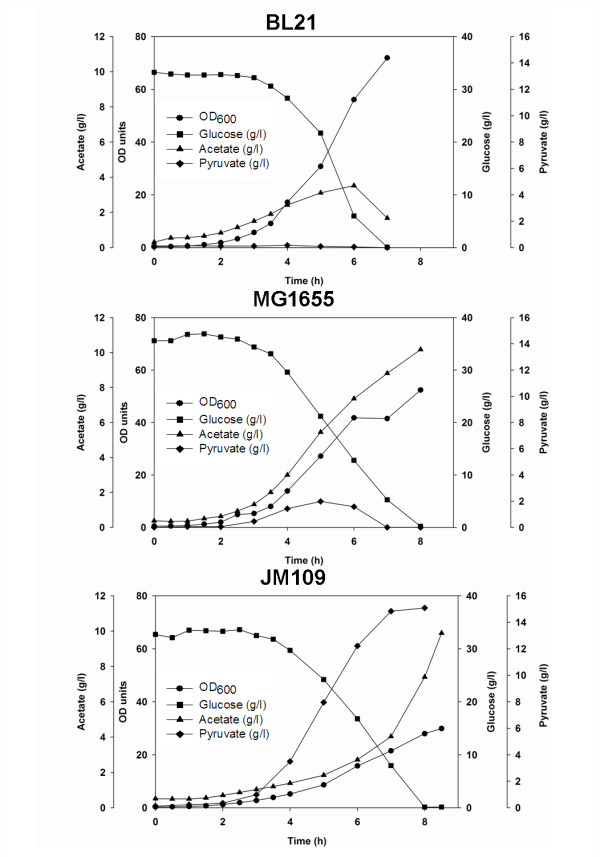
**Growth parameters of *E. coli *in bioreactor**. Growth of *E. coli *B (BL21), *E. coli *K-12 (MG1655) and *E. coli *K-12 (JM109) in a stirred tank bioreactor at 40 g/L Glucose.

**Figure 4:**
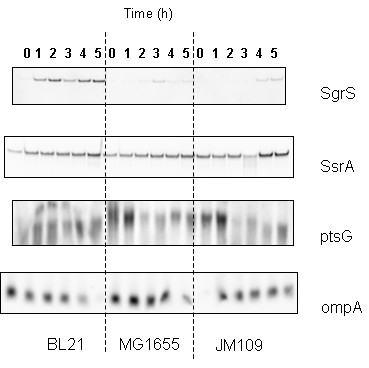
**Transcription levels of sRNA and mRNA from *E. coli *growing in bioreactor in high glucose concentration**. Transcription levels of *sgrS*, *ssrA*, *ptsG*, and *ompA *in *E. coli *B (BL21), *E. coli *K-12 (MG1655), and *E. coli *K-12 (JM109) in bioreactor at 40 g/L glucose

Following the shake flask growth, controlled growth of the three strains in a bioreactor at an initial glucose concentration of 40 g/L was performed. The growth profiles and transcription of *sgrS *and *ptsG *in *E. coli *B (BL21) and *E. coli *K-12 (MG1655 and JM109) are shown in Figure 3 and 4. Cell growth, glucose consumption, acetate and pyruvate production are shown in Figure 3. All strains showed similar glucose consumption pattern: *E. coli *B (BL21) grew more than *E. coli *K -12 (MG1655 and JM109), acetate production from *E. coli *K-12 (MG1655 and JM109) was around 10 g/L while *E. coli *B (BL21) produced only 4 g/L. Pyruvate concentration in *E. coli *K-12 (JM109) reached 15 g/L, compared with 2 g/L in *E. coli *K-12 (MG1655) and negligible concentration in *E. coli *B (BL21). The specific glucose consumption during the mid-log phase was ~0.6 gram per gram bacteria (wet weight) for *E. coli *B (BL21) and *E. coli *K-12 (MG1655), and ~1.3 gram per gram bacteria (wet weight) for *E. coli *K-12 (JM109). The corresponding transcriptions of *sgrS *and *ptsG *are shown in Figure 4. In *E. coli *K-12 (MG1655 and JM109) strains, no sRNA SgrS bands were detected during the first 3 hours of growth and faint bands were detected later; while higher transcription of SgrS was detected in *E. coli *B (BL21). The mRNA ptsG was observed in all strains, especially in the early growth stages, but lower transcription was observed in *E. coli *B (BL21). A decrease in the mRNA ptsG intensity was observed after 3 hours of growth, which may be due to the decreasing concentration of glucose in the media (Figure 3). The results obtained in the bioreactor were similar to those obtained in the shake flasks; the uniform detection of the internal controls SsrA and ompA in shake flasks and in the bioreactor indicated that the same amount of total RNA was loaded into the gel.

### Transcription of *sgrS *and *ptsG *in *E. coli *B (BL21) growing in the presence of low glucose in a bioreactor

**Figure 5:**
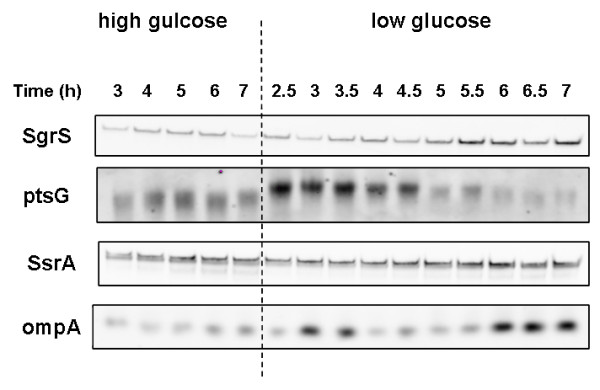
**Transcription levels of sRNA and mRNA from *E. coli *growing in bioreactor in low glucose concentration**. Transcription levels of *sgrS*, *ssrA*, *ptsG*, and *ompA *in *E. coli *B (BL21), in bioreactor at 40 g/L glucose (high glucose) and 1 g/L glucose (low glucose).

As indicated in the introduction, *E. coli *B (BL21) is not affected by glucose concentration, its acetate excretion levels are the same whether the bacteria is exposed to high or low glucose concentration. The transcriptions levels of *sgrS *and *ptsG *in this strain, growing in the presence of low glucose concentration, were measured to determine if the SgrS is involved in the control of the ptsG level. The results are seen in Figure 5. Comparing the transcription levels of *ptsG *and *sgrS *when the media contained 40 g/L glucose, with the transcription levels when the glucose concentration was kept around 1 g/L throughout the growth, showed that *sgrS *transcription were lower and *ptsG *transcription levels were higher.

## Discussion

Previous studies reported that increased levels of sRNA SgrS and reduced amounts of ptsG mRNA were observed in *E. coli *K-12 strains DJ480 PP6, IT1568, DH5α and W3110 when the bacteria were grown in the presence of the non-metabolizing αMG [11,13,15]. Reduced level of mRNA ptsG was also observed when *E. coli *K-12 was grown on glucose when the glycolytic pathway was blocked by mutation in phosphoglucose isomerase or phosphofructokinase [11]. The s*grS *transcription level was found to be correlated with the accumulation of αMG-phosphate or with G6P, and with the reduced level of the mRNA ptsG. The lower level of the mRNA ptsG was probably responsible for reduction in the biosynthesis of the glucose transporter IICB^Glc ^[13,16]. When *E. coli *K-12, DH5α and IT1568 were grown in the presence of 10 g/L glucose, sRNA SgrS was not observed, while transcription of *ptsG *was detected [14,15,17].

The results presented in this work confirmed the previously reported *E. coli *K-12 strains behavior in both shake flasks and in bioreactors, showing no sRNA SgrS or significantly low levels even when the glucose concentration was as high as 40 g/L. *E. coli *B (BL21) behaved similarly to *E. coli *K-12 (MG1655 and JM109) strains when αMG was added, but when the cells were exposed to 40 g/L glucose, unlike the *E. coli *K-12 (MG1655 and JM109) strains, *sgrS *was transcribed. When *E. coli *B (BL21) was grown in the presence of 1 g/L glucose, the SgrS level was lower and the ptsG level was higher, increasing the glucose transport into the cells. It is possible that this mechanism together with CRP-cAMP and Mlc controls the expression of the glucose transporter IICB^Glc^, thus reducing the internal glucose-phosphate concentration [18,19]. The *E. coli *B (BL21) responded to 40 g/L of glucose concentration and to the presence of αMG in the same way that *E. coli *K-12 (W3110) responded to αMG and to glucose when the glycolytic pathway was blocked [12].

The acetate production pattern of the tested strains when grown on 40 g/L glucose indicated better control of glucose assimilation in *E. coli *B (BL21) compared with *E. coli *K-12 (MG1655 and JM109) strains. This behavior is already known and is attributed to an active glyoxylate shunt, to higher flux through the TCA cycle, and to an active gluconeogensis pathway in *E. coli *B (BL21) [5]. The analysis of the SgrS-ptsG regulation suggests that the efficient intake of glucose and the lower production of pyruvate and acetate in *E. coli *B (BL21) are not only the result of different operation of the central carbon metabolism, but also the result of a different control of glucose transport into the cells. Since there is transcription of *sgrS *in *E. coli *B (BL21) and there is no transcription of *sgrS *in *E. coli *K-12 (JM109 and MG1165), there is less ptsG in *E. coli *K-12 (JM109 and MG1165) than in *E. coli *B(BL21). The SgrS-ptsG regulation likely contributes to lower concentrations of glucose and glucose intermediates in the cells, avoiding overloading of the TCA cycle and the accumulation of pyruvate and acetyl-CoA that can lead to acetate production.

The *sgrS *was transcribed in both *E. coli *B (BL21) and *E. coli *K-12 (MG1655 and JM109) strains when αMG was present in the media. But when both strains were grown in high glucose concentration, sRNA SgrS was detected only in *E. coli *B (BL21). If the trigger for the *sgrS *transcription is the accumulation of αMG-phosphate or glycolytic intermediates such as G6P as described previously [11,15], then it is possible that G6P or another intermediate of the glycolytic pathway accumulates in *E. coli *B (BL21) and does not accumulate in *E. coli *K-12 (JM109) or in *E. coli *K-12 (MG1655). The presence of pyruvate in *E. coli *K-12 (JM109) may be an indication that there is no accumulation of G6P, which is metabolized by the glycolytic pathway. Pyruvate and acetate accumulate as a result of limited availability of acetyl CoA by the pta-ack pathway. The information concerning the glucose consumption by *E. coli *B (BL21) and *E. coli *K-12 (JM109) strengthens the hypothesis that the difference in the acetate excretion between these two strains is related to the difference in glucose consumption [20]. *E. coli *MG1655 behavior is different; like *E. coli *K-12 (JM109), there is no *sgrS *transcription and there is acetate accumulation; but unlike *E. coli *K-12 (JM109), pyruvate accumulation is much lower [21]. It is possible that like in *E. coli *K-12 (JM109), G6P is not accumulated; rather it is being metabolized through the glycolytic pathway and contributes to the higher acetate concentration. It is more difficult to explain the *E. coli *K-12 (MG1655) behavior in response to pyruvate. It is also not certain that G6P is the trigger for the increase in the SgrS transcription in *E. coli *BL21 when it grows on high glucose concentration, as was the case when the glycolytic pathway was interrupted by mutation. It is possible that another intermediate or intermediates accumulate when *E. coli *B (BL21) grows at high glucose concentration and is responsible for the enhance transcription of the SgrS.

More work is required to understand the response of these microorganisms to the glucose concentration. Based on these results, our assumption is that the difference in the glucose transport is the result of the lower ptsG mRNA amount which contributes to lower concentration of the glucose transporter IICB^Glc^. The control of the glucose transporter concentration is possibly another mechanism in *E. coli B *(BL21) which helps reducing the overflow of glucose and to minimize acetate production.

## Conclusions

SgrS is a small RNA in *E. coli *which regulates the expression of the glucose transporter IICB^Glc ^and hence the glucose uptake by the cells. *sgrS *transcription was found to be regulated differently in *E. coli *B (BL21) and *E. coli *K-12 (JM109 and MG1655) when the cells were exposed to high glucose concentration. In *E. coli *K-12, *sgrS *transcription was lower which contributes to higher transporter concentration and to higher glucose uptake. In *E. coli *B (BL21) the situation was the opposite; *sgrS *transcription was higher which leads to lower glucose transporter concentration and to lower glucose uptake. It was reported earlier that the reason for increased *sgrS *transcription may be related to glucose phosphates accumulation; this was shown when *E. coli *K-12 (JM109 and MG1655) was exposed to αMG or when the glycolysis pathway was blocked. We therefore suggest that the reason for the higher *sgrS *transcription in *E. coli *B (BL21) is related to accumulation of an intermediate or intermediates of the glycolytic pathway such as G6P which serves as an indication for a possible overflow of glucose. This situation is different in *E. coli *K-12 (JM109 and MG1655) where, possibly, there is no accumulation of an intermediate of the glycolytic pathway and the glucose is converted to acetate and pyruvate. The above suggested mechanism contributes to the lower acetate production in *E. coli *B (BL21) when growing at high glucose concentration. The central carbon metabolism in *E. coli *B (BL21) is known to be more suitable to handle high glucose concentration as a result of its higher TCA flux and active gluconeogensis and glyxoyalte pathway. The control of the glucose transport into the cells is another tool in this bacterial strain to minimize the overflow of glucose and minimize acetate production.

## Materials and methods

### *E. coli *strains and growth conditions

*E. coli *K-12 (MG1655) (F-, λ-, ilvG-, rfb-50, rph-1); *E. coli *B BL21(DE3) (F-, ompT, hsdSB(rB-, mB-), gal, dcm (DE3)) and *E. coli *K-12 (JM109(DE3)) (endA1, recA1, gyrA96, thi, hsdR17(rk-, mk+), relA1, supE44, λ-, _(lac-proAB), [F', traD36, proAB, laclqZ_M15], IDE3) (Promega Corp, Madison, WI) were used. Cells were grown at 37°C in modified LB medium containing 10 g/L tryptone, 5 g/L peptone, 5 g/L yeast extract (15 g/L for JM109), 5 g/L NaCl and 5 g/L K_2_HPO_4_. After sterilization, 10 mM MgSO4 and 1 ml/L trace metal solution were added. Overnight cultures were used to inoculate shake flasks at OD600 = 0.03. Shake flasks were incubated at 37°C with constant agitation. When cells reached an OD600 of approximately 0.3, 20 g/L glucose or 1% αMG was added. After glucose or αMG addition, samples were collected for sRNA and mRNA analysis. Cell culture was centrifuged at 13,000 g for 5 min; the cell pellet and the supernatant for RNA extraction and metabolites analysis were maintained at -80°C. For bioreactor experiments, a 5 liters B. Braun fermentor equipped with data acquisition and adaptive dissolved oxygen control system was used. Following sterilization and supplement addition, the glucose concentration was adjusted to 40 g/L. pH was controlled at 7.0 by addition of 15% (w/v) NH_4_OH, and dissolved oxygen (DO) was controlled at 30% of air saturation. The bioreactor was inoculated with OD_600 _of 0.3 and samples were collected and processed as described for shake flasks. Cell density was determined by measuring OD600 with a Pharmacia Biotech Ultrospec 3000 UV/Visible spectrophotometer. For the low glucose concentration experiment, glucose was maintained at 1 g/L by using DO control [22].

### Metabolite analysis

Glucose was determined by YSI 2700 SELECT Biochemistry Analyzer. Acetate and pyruvate were analyzed by HPLC, Hewlett Packard 1100 Series using Aminex resin-based HPX-87 H column (Bio-Rad). Separation conditions were as follows: wavelength 210 nm; mobile phase 0.008 N H_2_SO_4_, flow rate 0.6 mL/min, temp 35°C for acetate and 60°C for pyruvate. Three standards with concentrations of 0.1 g/L, 1.0 g/L, and 10 g/L were used for the calibration curve. The retention time for the pyruvate was 10 min and for the acetate 14 min.

### RNA extraction

For RNA extraction, the hot phenol method was used: cell pellets were resuspended in 0.5% SDS, 20 mM NaAc, and 10 mM EDTA and extracted twice with hot acid phenol:chloroform followed by two extractions with phenol:chlorform isoamyl alcohol. Absolute ethanol was added and the extract was kept at -80°C for 15 min. After centrifugation at 14,000 g for 15 min, the pellets were washed in 70% ethanol. RNA was air dried and resuspended in ultrapure water (KD medical USA). RNA was quantified using NanoDrop 1000 spectrophotometer (Thermofisher Scientific).

### Northern blot analysis

Northern blot analyses to detect sRNA SgrS were performed as described previously [13]. 5 μg of total RNA was run on a TBE 10% urea polyacrylamide gel and transferred to a positively charged nylon membrane. A 5'-biotinylated oligonucleotide SgrS-specific probe and Bright-Star Biodetect non-isotopic kit (Ambion, Inc.) were used for detection. For detection of mRNA ptsG, 5 μg of total RNA was run on a 1.2% denaturing agarose gel and transferred to a positively charged nylon membrane [23]. After hybridization, the washed membranes were conjugated with streptavidin-alkaline phosphatase following the BrightStar Biodetect Kit (Ambion). Chemiluminscent signals were detected using the Fujifilm LAS-4000 imaging system. The image before the saturation point was recorded. The internal controls SsrA and ompA were detected by probing again the stripped membranes with boiling in 0.5% SDS. The sequences 5' to 3' of the probes used were:

*sgrS *(Bio)-GCAACCAGCACAACTTCGCTGTCGCGGTAAAATAGTG

*ptsG *(Bio)-CAGCCAGCTGAAATTCGCGGAACCGACGCCCAGCAG

*ssrA *(Bio)-CGCCACTAACAAACTAGCCTGATTAAGTTTTAACGCTTCA

*ompA *(Bio)-CCATTGTTGTTGATGAAACCAGTGTCATGGTACTGGGACCAGC

## List of abbreviations

CRP-CAMP: transcriptional dual regulator; MLC: global regulator of sugar metabolism; *OMPA*: messenger RNA encoding outer membrane protein used as internal control; *PTSG*: gene encoding for the polypeptide ptsG; PTSG: polypeptide ptsG; *SGRS*: gene encoding for the sugar transport related small RNA; SGRT: peptide SgrT; SGRS: sugar transport related small RNA; SRNA: small RNA; *SSRA*: small RNA encoding transcription unit used as internal control.

## Competing interests

The authors declare that they have no competing interests.

## Authors' contributions

AN participated in the design of study, fermentations and molecular analysis, analyzed the results and drafted the manuscript. WN participated in the fermentations and the molecular analysis. YS conceived the study, participated in its design and coordination, and in the writing of the manuscript. All authors read and approved the final manuscript.
